# 

**Published:** 1864-11

**Authors:** 


### CHICAGO MEDICAL JOURNAL.

**Terms, $2.00 per Annum.**

### CONTENTS OF THE NOVEMBER NUMBER.

PAGE.

ORIGINAL COMMUNICATIONS.

Address Introductory to the Twenty-Second Annual Course of Lectures in Rush Medical College. By De Laskie Miller, M. D., Professor of Obstetrics and Diseases of Women and Children .................... 479

Report on Orthopedic Surgery, made at the Annual Meeting of the Illinois State Medical Society, convened in Chicago, May 3d, 1864. By David Prince, M. D., Jacksonville, Ill. (Continued) .................... 495

Clinical Lectures on Diseases of the Eye. By E. L. Holmes, M. D., of Chicago, Lecturer, on Diseases of the Eye and Ear in Rush Medical College—Iritis .................... 509

SELECTED.

Complicated Case of Labor. Prolapsus of the Funis, etc .................... 514

Editorial and Miscellaneous .................... 519

Proceedings of the Galen Medical Society, of Stark Co., Ind .................... 522

Neuralgia over the Spine of certain Vertebræ .................... 524

New Anæsthetics .................... 525

New Use of Electricity .................... 525

